# Development of a chest X-ray machine learning convolutional neural network model on a budget and using artificial intelligence explainability techniques to analyze patterns of machine learning inference

**DOI:** 10.1093/jamiaopen/ooae035

**Published:** 2024-05-02

**Authors:** Stephen B Lee

**Affiliations:** Division of Infectious Diseases, Department of Medicine, College of Medicine, University of Saskatchewan, Regina, S4P 0W5, Canada

**Keywords:** development, chest X-ray, classification, model, budget, SHAP

## Abstract

**Objective:**

Machine learning (ML) will have a large impact on medicine and accessibility is important. This study’s model was used to explore various concepts including how varying features of a model impacted behavior.

**Materials and Methods:**

This study built an ML model that classified chest X-rays as normal or abnormal by using ResNet50 as a base with transfer learning. A contrast enhancement mechanism was implemented to improve performance. After training with a dataset of publicly available chest radiographs, performance metrics were determined with a test set. The ResNet50 base was substituted with deeper architectures (ResNet101/152) and visualization methods used to help determine patterns of inference.

**Results:**

Performance metrics were an accuracy of 79%, recall 69%, precision 96%, and area under the curve of 0.9023. Accuracy improved to 82% and recall to 74% with contrast enhancement. When visualization methods were applied and the ratio of pixels used for inference measured, deeper architectures resulted in the model using larger portions of the image for inference as compared to ResNet50.

**Discussion:**

The model performed on par with many existing models despite consumer-grade hardware and smaller datasets. Individual models vary thus a single model’s explainability may not be generalizable. Therefore, this study varied architecture and studied patterns of inference. With deeper ResNet architectures, the machine used larger portions of the image to make decisions.

**Conclusion:**

An example using a custom model showed that AI (Artificial Intelligence) can be accessible on consumer-grade hardware, and it also demonstrated an example of studying themes of ML explainability by varying ResNet architectures.

## Background and significance

Machine learning (ML) is poised to make large impacts in healthcare, advancing medical care but also fundamentally changing and improving our existing delivery of care. ML applications have been developed that perform at or even exceed human capacity.^1^^,^^2^ However, there are unique challenges and considerations that remain unresolved.

Traditionally AI requires significant computational resources,^3^ which may have led to a digital divide in ML research.^4^ Generation of large datasets may be linked to research, potentially causing a compounding effect.^5^ Approaches enhancing accessibility of AI work could support broader engagement.^4^^,^^6^

While other fields have seen enormous changes from AI, ranging from self-driving cars to chatbots and fraud detection, healthcare has been understandably slower due to confidentiality, logistic, and privacy concerns. It is challenging to curate massive scale, granular databases in healthcare. Much existing work has been on specific tasks, such as identifying infections on chest radiographs (CXR).^7^ However, generalized tasks are significantly more difficult and have often required massive datasets.^3^ For instance, CheXNet (based on Densenet-121) has been employed by numerous authors to detect abnormalities on CXR.^8^^,^^9^ Other model architectures such as Inception V3, VGG16, and even ResNet have been employed in the past.^10^^,^^11^

Furthermore, while knowledge of the advancement of ML in the technology industry is widespread, ML is a topic where many clinicians are less knowledgeable.^12–14^ Consequently, many may not be aware that creation of certain ML models can be accomplished with consumer-level resources. Very few manuscripts in the medical domain discuss the hardware required for ML work and of these most used professional hardware.^15–18^ This study attempts to perform ML work with publicly available datasets and consumer grade hardware.

Additionally, while convolutional neural networks (CNNs) have been used for radiography ML models, work is still undergoing as to optimal architectures and pre-processing steps. Histogram equalization has been explored as a technique for contrast enhancement in chest radiography studies; however, most of this work has focused on specific conditions, with the vast majority in COVID-19.^19^^,^^20^ However, its use in a generalized rather than focused task helps researchers better understand the potential applications of contrast enhancement.

Finally, a concern in AI is that the processes by which a machine makes inferences are often unknown. This black box is unsettling as there may be inherent flaws in performance or unintended bias. In its guidance for healthcare AI, the WHO has also called for AI to be explainable.^6^ Recently, numerous visualization methods have emerged to help deduce reasoning in ML models.^21^^,^^22^ However, each ML model is different and conclusions on 1 model do not necessarily translate to others. Indeed, each instance of training, even in the same model, is inherently stochastic. Thus, much like individual humans, the way 1 model makes inferences is not translatable to another. While studies on AI explainability have begun to emerge, they have focused on individual models and not on larger patterns and themes. A systematic way of studying concepts in inference generation would help us better understand ML.

While using ML for radiographs has been previously reported, this study explores various concepts through development of an example ML model (CNN to classify CXR as normal or abnormal). This work’s insights will help future healthcare researchers in ML studies.

## Objective

This study creates an ML model, a CNN trained to classify CXR as normal/abnormal. Through the development of this model, it explores various concepts. Firstly, it creates and trains this model using fully publicly available datasets and consumer-grade hardware. The intent was not to create the highest performing model in literature, but rather to demonstrate the feasibility of doing this without elite-level resources. Secondly, it explores a mechanism of contrast enhancement that could be useful in improving the radiograph classification performance. Finally, the study explores the idea of using AI explainability to analyze overarching thematic elements of how models make inferences (rather than individual performance).

## Methods

### Training data and hardware

As an objective was to utilize widely available hardware, the entire work, including data pre-processing and training, was performed using a consumer-grade personal computer. Some pre-processing used a central processor unit; however, most tasks including training utilized a single graphics processing unit (GPU) (eVGA nVidia 3090).

A second study objective was to demonstrate the ability to use publicly available, limited datasets to train an operational ML classification model. As such datasets were obtained within Kaggle.

As the intent of this work was to demonstrate the ability to make ML more accessible and to use publicly available datasets, limitations of these public datasets had to be accounted for. Many publicly available datasets were not large enough or diverse enough to provide robust training to the model. Therefore, 3 datasets were combined to help improve our model’s performance. A subset of the NIH (National Institutes of Health) Chest X-ray Dataset was used with proper classification.^23^^,^^24^ These images were combined with the publicly available COVID Qu-Ex Dataset^25–29^ and Tuberculosis (TB) Chest X-Ray Database.^30^ These 2 datasets were chosen as they were publicly available and contained large volumes of clinically accurate data. However, given they focused on specific conditions, it was important to ensure those diagnoses were not over-represented which would have led to overfitting. Thus, only a selection of the COVID-19 and TB data was chosen. The specific amount taken was a process of trial and error through multiple iterations of model development. A total of 2509 images were used and are available on GitHub (https://github.com/leestephenz/cxrproject/).

### Machine learning model and development

**Figure 1.**
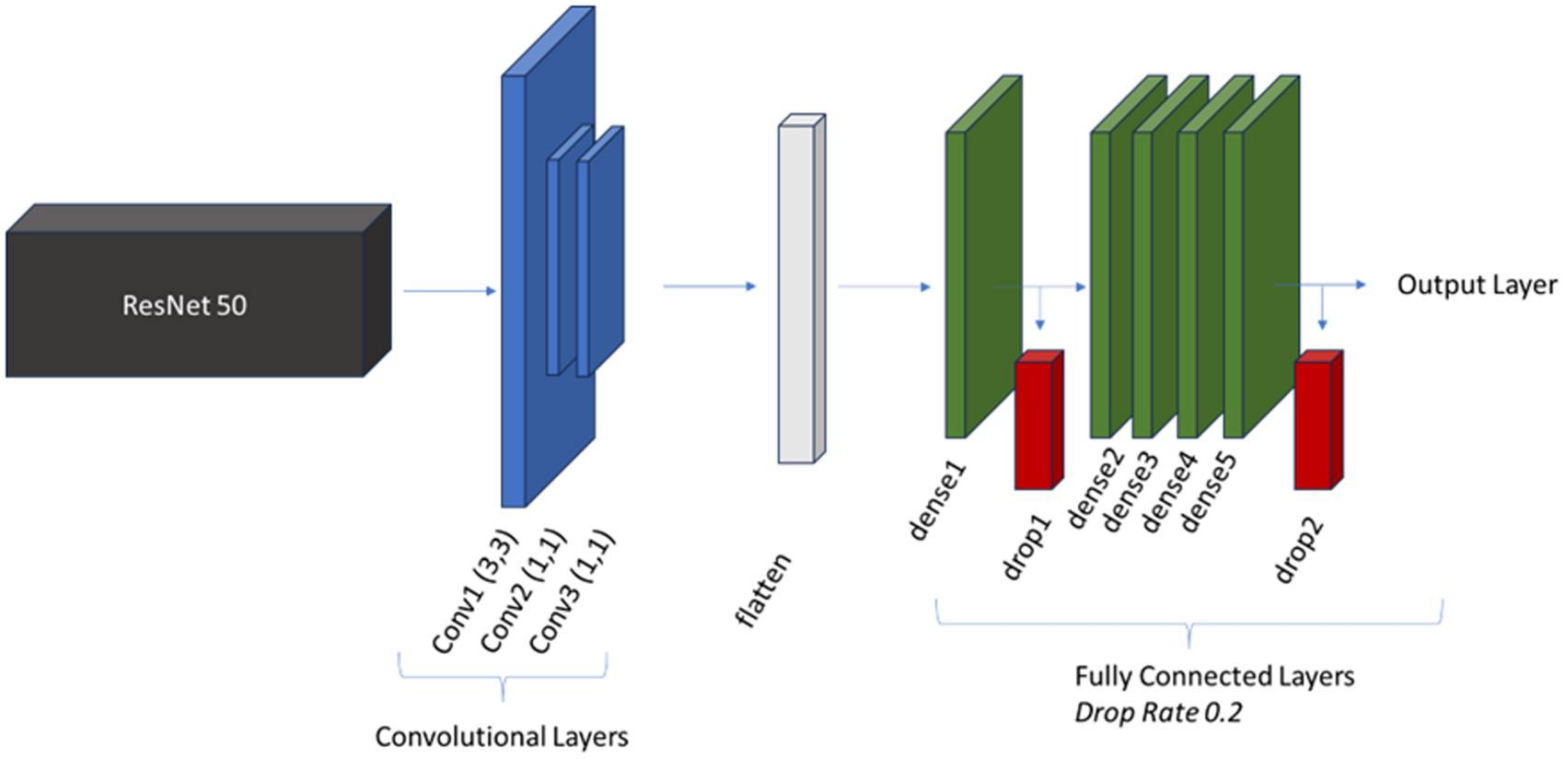
Model architecture.

The model was trained and built with Python 3.9.16 and TensorFlow 2.6.0. The model created used transfer learning. ResNet50 was chosen as the base model. Additional custom layers were added to ResNet50 and hyperparameters were tuned based on this architecture to maximize our test statistics. Some tuning, such as batch sizing and filters in custom layers, were limited by hardware capabilities. The architecture of this study’s model is seen in Figure 1. Vanishing gradients is a problem that arises when building deep neural networks which may be required with advanced computing tasks. ResNet is an architecture designed to solve the vanishing gradient problem through the concept of skip connections that bypass layers, first described by He et al.^31^

The convolutional layers used 32 filters each with other parameters seen in Figure 1. In these layers, the scaled exponential linear unit (SELU) activation function was chosen, as it was found to lead to faster convergence in training and better performance when compared to other activation functions trialed. In the fully connected layers, 64 filters were used, and the traditional rectified linear unit (ReLU) activation function was chosen. In testing, 2 drop layers in the fully connected layers helped prevent overfitting and increased the ability for the model to generalize in the test set. Finally, an output layer with a sigmoid activation for binary classification determined whether the CXR was coded as normal or abnormal.

An adaptive moment estimation (Adam) optimizer was chosen and hyperparameter tuning performed for the decay steps and rate. A learning rate of 0.001 was used with exponential decay and a batch size of 32 used.

The development of the model was iterative in nature, as is typical in ML work.^32^ During the coding process, multiple errors were encountered which required code debugging and iterations improved final model performance. While unfeasible to report the entire log of debugging, code improvements, data cleaning, and hyperparameter tuning iterations, there were significant milestones in development. One of the largest milestones was a clinical review of the datasets. The initial version of the NIH Chest X-ray dataset resulted in low model performance (∼50% accuracy) and upon manual review by the clinician author, it was discovered the original NIH Chest X-ray dataset was erroneous. A smaller modified version (available on Kaggle) where images were read by 5 American Board-certified radiologists was used.^23^^,^^24^ Other milestones included hyperparameter tuning and model modifications, of which the most significant were modifications to batch size, epoch cycles, neurons per layer, total layers, and the addition of drop layers. The nVidia 3090 had 24 GB of memory and thus did have limitations in data processing. As such different permutations of batch size, neurons per layer, and number of layers were performed to balance overfitting, model performance, and number of parameters (too many parameters caused kernel failure due to overwhelmed memory). Drop layers were added and reduced overfitting. Too little epoch cycles would result in a lack of convergence (the model did not have enough cycles to learn) while too many epoch cycles resulted in overfitting.

Finally in accordance with best practices, a review of existing appropriate frameworks was performed,^33^ and the most appropriate framework chosen for this model^34^ (Table S1).

### Data processing, testing, and contrast enhancement module

The full details of our code, including pre-processing and our machine learning model are available for re-use or access on GitHub. Data was split into training and testing; a separate section of code was written for model testing and test images were never seen by the model during training. This separation ensured the model performance was not confounded. The images presented to evaluate performance were completely novel. A total of 464 images were used for testing and were a novel sub-section of the images previously mentioned. It was composed of images from all 3 sources (NIH Chest X-ray, COVID Qu-Ex, TB). Based on the pairing of labels with images, there was no missing data (GitHub).

In clinical encounters, when an image has subtle abnormalities, human clinicians may use contrast-enhancement techniques (computer tools with digitized CXRs or using light boxes prior computerization^35^) As such, a similar process was built into our model, using a contrast enhancement module during the testing phase. As clinically it is important to identify abnormalities, it was deemed important to improve sensitivity (recall), so this contrast module was applied only to those classified as normal.

For images where the prediction of the machine was greater than 0.3 but less than 0.49999 (images that came close to being abnormal), the machine would use histogram equalization.^36^ After modification of the images, it would then put these back through the model and re-run the predictions.

### Explainable AI

This study used the Shapeley Additive exPlanations (SHAP) method to help interpret the machine’s inferences.^37^

As different models and even instances can cause differing SHAP heatmaps, to help better understand AI in general, this study attempted to characterize patterns of inference generation based on characteristics of architecture. The base model was changed from ResNet50 to ResNet101 and ResNet152 to determine what effects changing depth would have on the SHAP heatmaps. All other elements stayed the same and the concept driving the architecture of the base model (ResNet’s skip blocks) remained the same. Each increasing ResNet architecture contained the corresponding number of layers (50, 101, and 152, respectively). Furthermore, increasing architecture also increased the number of residual blocks with ResNet50 starting at 16.^38^ In summary, each higher level ResNet added depth and complexity.

To quantify the exact number of pixels used, custom code was constructed to quantify the number of colored pixels as a percentage of the total pixels (available on GitHub).

## Results

The primary model (custom CNN built on top of ResNet 50) achieved an accuracy of 0.7866, recall of 0.6873, precision of 0.9615, and an AUC of 0.9023. When the contrast enhancement was applied, the accuracy improved to 0.8190 and recall to 0.7423 (Table 1).

**Table 1.** Test performance on various models.

Model	Accuracy	Recall	Precision	AUC
Custom model (ResNet50 Base)	0.7866	0.6873	0.9615	0.9023
With contrast enhancement	0.8190	0.7423	0.9600	0.9020
Custom model (ResNet101 Base)	0.7845	0.7113	0.9283	0.8823
With contrast enhancement	0.7931	0.7354	0.9185	0.8834
Custom model (ResNet152 Base)	0.7672	0.6667	0.9463	0.8664
With contrast enhancement	0.7672	0.6667	0.9463	0.8724
ResNet50 Base (no additional layers or training)	0.3685	0.0103	0.3750	0.2815
With contrast enhancement	0.3685	0.0103	0.3750	0.2812

Directly comparing this study’s model to ResNet50 alone (without customized additions) was not possible as ResNet50 was designed to identify multiple different classes of non-medical images rather than a binary outcome. However, to approximate ResNet50’s un-modified performance, a single flatten and fully connected output layer was added. The model was not trained and simply ran on the test dataset. Our model performed significantly better than this base ResNet50 with minimal modifications (Table 1).

To help elucidate the effect of deeper ResNet architectures, we substituted the ResNet50 base with ResNet101 and ResNet152. Hyperparameters were tuned for our primary model, the custom CNN built on ResNet50, and to maintain consistency, they were not modified for the deeper architectures. Results are seen in Table 1.

**Figure 2.**
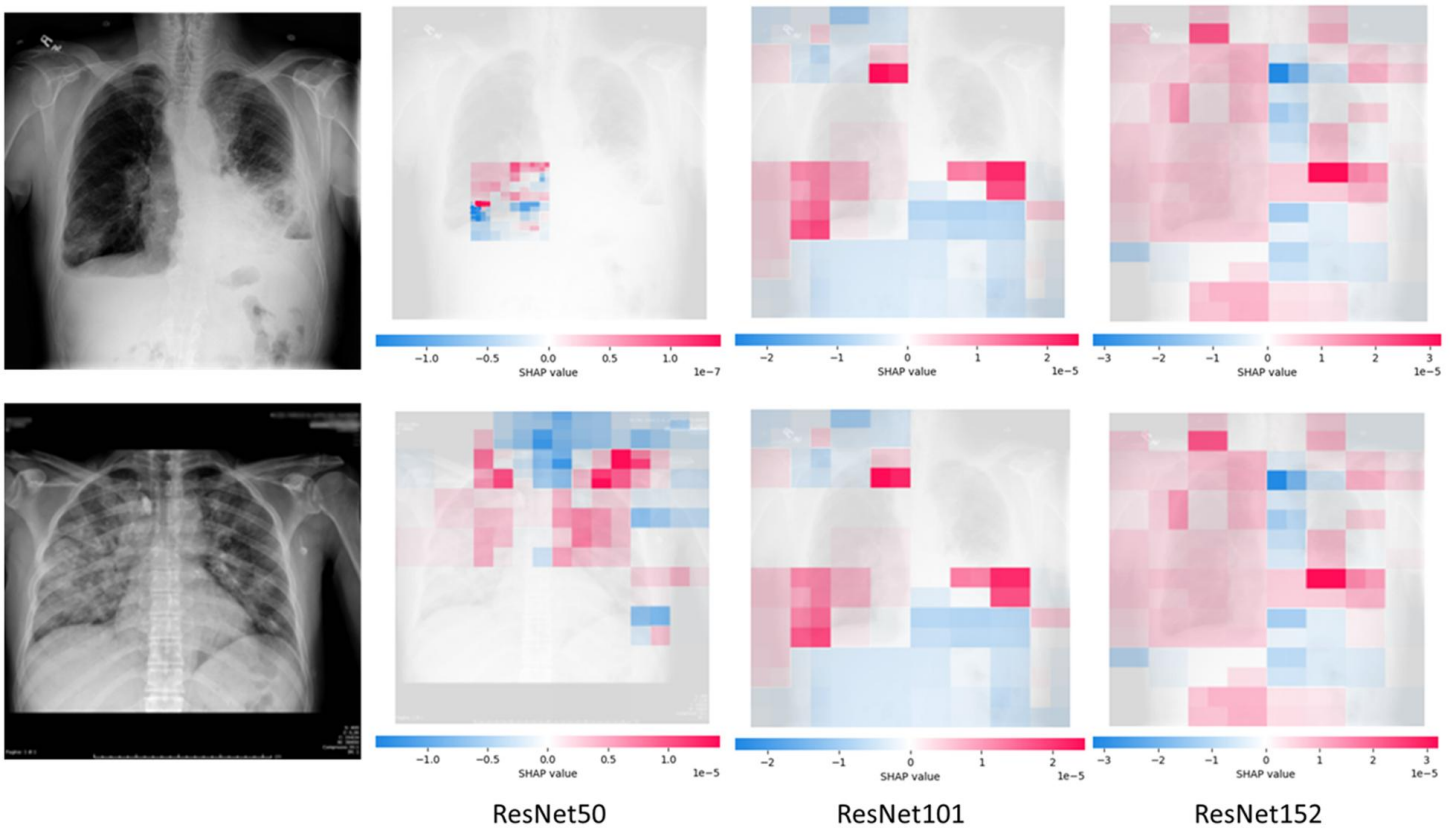
SHAP images for CXR Inference, ResNet50, ResNet101, ResNet152 base model.

SHAP values were run across the images to attempt to find patterns in machine inference. This study looked at the SHAP values for our primary model but also how this changed based on deeper base models (Figure 2). As the ResNet base model became deeper, more of the image was used to make an inference.

The percentage of colored pixels, thus the number of pixels used in inference, increased based on the depth of the ResNet model. For the first CXR, 6.28% was used by ResNet50, 67.48% by ResNet101, and 87.69% by ResNet152. For the second CXR, 56.40% was used by ResNet50, 83.57% by ResNet101, and 96.99% by ResNet152.

## Discussion

This study created a model that displayed an ability to properly classify CXR as normal or abnormal across a wide range of conditions (Accuracy 79%, Precision: 96%, Recall 69%, AUC 0.9023; Post-Contrast Adjustment Accuracy 82%, Precision 96%, Recall 74%, AUC 0.9020). While our model does not outperform respirologists and radiologists, it does outperform some clinicians.^39^ Direct comparisons are difficult as other models often focus on targeted rather than general tasks, but this model has a comparable performance to many works.^9^^,^^11^^,^^40^ Regardless we primarily use this model to explore ML concepts rather than focusing on its performance.

In clinical practice, a technique used by clinicians involves increasing contrast to visualize subtleties.^35^ Similarly, this study used histogram equalization in a post-processing step whereby borderline CXRs below the threshold underwent contrast enhancement and were re-fed through the predictive model.^36^ Using this human analogous process, performance increased slightly (Accuracy 82%, Precision 96%, Recall 74%, AUC 0.9020). While described in general imaging tasks, such methods are not as often reported for general CXR tasks and warrant future clinical study. While our modification improved recall by decreasing false negatives, a similar technique could be applied to precision by decreasing false positives.

While ML is poised to make a big impact in healthcare, it may create inequities due to resource disparities.^4^^,^^41^ Traditional ML models created by industry have massive datasets.^42–44^ In healthcare, this is difficult given multiple limitations such as privacy, logistics, lack of data science knowledge, and limited pre-trained models. Furthermore, not all institutions have capabilities of curating massive scale datasets, and those that do, do not share publicly. A study showed that there is a bias in research productivity and datasets.^5^ This limitation has traditionally formed a barrier to the advancement of healthcare ML.

This study used a dataset of 2509 images with consumer hardware, one GeForce 3090 RTX, to achieve a reasonable performance on a model that classified CXRs as normal or abnormal across a variety of generalized abnormalities (including hernias, pulmonary, and cardiac abnormalities). To achieve our metrics, we utilized transfer learning combined with our own custom CNN. ResNet50 (public package on Python) was a general image recognition model trained on everyday images.^31^ Our additional steps improved model performance on CXRs. The base performance of the ResNet50 model without further layers or training was significantly lower than the custom model (Table 1). The transfer learning of ResNet pre-taught our model general recognition tasks which lowered overall computational and dataset requirements.

Recognizing the importance of transparency as highlighted in multiple frameworks,^33^ this study provides a case for the viability of consumer-grade hardware and public datasets in healthcare ML studies. While the power of consumer-grade hardware is known within the technology community, few medical studies have reported specific hardware used.^15–18^

Future work could investigate the role of transfer learning to help adapt trained models to under-represented populations. If existing models trained in areas with over-representation in research are found to have poor generalizability, transfer learning combined with additional architecture or datasets could help. This could circumvent limitations of building new models from scratch with small datasets.

CNNs have traditionally been thought of as black boxes because the features a machine uses to make inferences are not readily decipherable by humans.^45^ This uncertainty has led many to describe inadvertent risks of ML. Gichoya et al showed that an ML model was able to differentiate patient race on radiographs. It persevered despite image manipulation and the researchers could not determine how the machine made its inferences. ML has great potential for improving healthcare; however, it is not without risks. Despite these risks, ML integration in medicine will be inevitable. We must find ways to systematically study machine learning inference.

However, other models did not have similar concerns.^46^ This difference illustrates that individual architectures, models, and even instances behave differently: an over-arching conclusion cannot be reached regarding ML. Each model’s training process and each instance of training is stochastic, thus is not comparable across studies. For instance, one cannot say that all AIs recognize pleural effusions by looking at blunting of the costovertebral angle, rather only that a specific model recognizes pleural effusions via angle blunting. Due to these variations, an approach where we, instead, look at types of models and patterns of inference may be useful in helping explain AI.

While directly understanding the function of each neuron or layer is not feasible, there exist indirect methods of observing a machine’s attention.^47^ ResNet’s architecture was designed around the concept of a skip connection, which tackles vanishing gradients in deep networks.^31^ There are variations of ResNet based on the number of layers it contains. While this study’s primary model was built around and tuned with ResNet50 (50 layers) as the base for transfer learning, ResNet50 was substituted with ResNet101 and ResNet152 to determine what effect deeper ResNet architectures may have on machine inference. ResNet101/152 are sequentially deeper and more complex, characterized by increasing number of layers and residual blocks.^31^ In all cases the additional custom CNN layers and hyperparameters remained consistent. SHAP was used to determine which areas of the image the machine focused on, and how each area it focused on affected its classification. These images were compared across the three base architectures. It was found that, like human clinicians, the machine focused on lung fields when looking at CXRs to classify them. It generally looked at areas of high contrast (opacities against the black background). These findings mimic those of a study looking at breast density where for correct predictions, the machine would focus its attention on areas that also seem intuitive to humans.^48^

Such AI explainability methods are important as without them, it is possible the model is using an aberrant method of inference. For instance, if certain hospitals had more abnormal CXRs, the model could end up using a hospital image watermark to predict abnormality. Using SHAP helped us increase our confidence that the model behaved properly.

While this is important, the use of SHAP has already been reported.^49^ What is unique about this study is that we use SHAP not to explain the behavior of a specific model, but rather, it studied how varying a specific feature or characteristic of a model impacts its behavior. An analogy could be drawn to the neurosciences, where functional magnetic resonance (fMRI) may be used to study the cortical activation of various tasks.^50^ Rather than looking at the fMRI of a single brain, one could look at the effect a temporal lobe has on cognition in general.

This study found that the deeper the ResNet-base architecture, the wider focus the machine employed. All the models were supplied with the entire image. In the ResNet50 images, the machine would focus on specific quadrants. However, with ResNet101/152 the machine showed attention to multiple quadrants or even the entire CXR (evidenced by increasing ratio of colored to non-colored pixels). Of note a comparison of ResNet50/101/152 performance is not possible as hyperparameters were tuned for ResNet50 and equal tuning could have resulted in benefits.

This use is novel and important as each model and even each instance of a model can vary due to different instantiations. AI explainability conclusions from a single model cannot be extrapolated to a wider understanding of ML. However, by studying thematic elements, such as varying depths of ResNet on behaviour, researchers may achieve better understanding of ML in general. Indeed, experts have advocated for a hierarchy of understanding AI, from transparency to explainability.^51^ This study’s approach to studying thematic elements of architecture could be a different paradigm for studying ML behavior and help us achieve a higher level of understanding how ML functions. This would be in an analogous fashion to understanding general psychological principles or behavioral patterns as compared to understanding the cognition or decisions of a single individual.

Understanding ML is key to ensuring we build models that do not have aberrant inference processes; thus, the greatest benefit of thematic work is a new way to study inference. However, this work also has concrete benefits. Traditionally AI explainability methods have been applied after model completion. However, if general rules apply to various architectures, researchers can make choices even before model construction; for instance, choosing a deeper ResNet architecture if one desires larger parts of an image to be used in inference. This could reduce resources and time required for model development. Furthermore, it also establishes a foundational methodology for broader exploratory research in AI. Future work could analyze other themes in ML work. An example could be the differences in inferencing behavior between DenseNet^8^^,^^9^ and ResNet^31^ architectures or the effect of the residual block itself. This could lead to the development of methods for forward-looking prediction of behavior.

A series of limitations exists with this study. ML models differ from each other. Performance and inference change based on the set of training data that models are exposed to, hyperparameter settings, or even per instance of training (initialization of model weights and biases are random). Thus, findings cannot be extrapolated literally, and performance may not be consistent with other datasets. Specifically, it is noted that the process of model development and training is inherently stochastic, the reported version is one of the higher performing iterations, but it is possible that performance estimates are optimistic and not generalizable. While the intent was to demonstrate accessibility of AI using consumer grade hardware and public datasets, true democratization requires internet access and advanced data science skillsets. In healthcare it also requires considerations of patient privacy and dealing with the challenges of nuanced tasks. However, this study demonstrates that consumer grade hardware and lack of access to massive scale data does not inherently form an absolute barrier.

A particular limitation was the nature of publicly curated datasets. While such datasets have led to significant progress in ML, often they do not have detailed methodological descriptions in the curation process. It is vital that models deployed in clinical settings be tested rigorously with multiple datasets. While it may not be possible to train models in the development phase with such data, testing could occur with highly validated privately curated datasets from hospital authorities. Furthermore, this limitation underscores the need for high quality data to progress healthcare ML.

The intention of this study is less model performance, rather the use of the model to explore concepts, such as the ML work accessibility, contrast enhancement, and finally an example of studying themes in AI explainability rather than individual models. It is limited in that it is based off one model and future detailed work could be performed on each concept independently.

## Conclusion

Using a novel CXR classification model, this study demonstrated the possibility of performing medical studies on consumer-grade hardware using public datasets, employed contrast enhancement, and demonstrated using AI explainability methods in a novel fashion, by looking at thematic elements of models (varying ResNet depths) rather than an individual model’s behavior. By doing so, it determined that increasing ResNet depths used increased inference area. This technique could be used to help researchers better understand ML behavior. This work explored concepts that could be studied in future studies with different models and tasks.

## Supplementary Material

ooae035_Supplementary_Data

## Data Availability

Data is available and free to use through GitHub and Kaggle.
