# Supplementary material for: Development of a chest X-ray machine learning convolutional neural network model on a budget and using artificial intelligence explainability techniques to analyze patterns of machine learning inference
Source: JAMIA Open. 2024 May 2;7(2):ooae035. doi: 10.1093/jamiaopen/ooae035 (PMC11064095; doi:10.1093/jamiaopen/ooae035)
Supplement: ooae035_Supplementary_Data [file ooae035_supplementary_data.docx]

*Supplementary Table 1. Framework for Chest X-Ray Classification Machine Learning Model*

| Question | Response Applied to this Model |
| --- | --- |
| What is the health question relating to patient benefit? | The benefit is not directly clinical, the model is not intended for any clinical deployment. However, its benefits are indirect. The intent of this paper is to demonstrate potential feasibility of ML development on consumer hardware with public datasets. Furthermore, the insights it reveals regarding various pre-processing methods and thematic analysis of AI explainability may help future ML models designed for clinical practice. These benefits would indirectly help patients. |
| What evidence is there that the development of the algorithm was informed by best practices in clinical research and epidemiological study design? | Best practices were performed as applicable to this study. The work was guided by a clinician author. Only data that was already publicly available were used and no patient identifiers were included to maintain ethics. The methodological goals of the paper were set out prior to development. As diverse a dataset as possible (given constraints of focusing only on public datasets) was created and three different datasets were purposely mixed which comprised images from a diverse set of nations. The origins of data were not always reported by authors, but it comprised at least the United States, Europe, and China. It represented a multisite data source, thus lowering bias and improving generalizability. External validation was performed using a test set not seen during the training process. All data and code were made publicly available for interest of transparency. |
| When and how should patients be involved in data collection, analysis, deployment, and use? | Public datasets were already created, and engagement of patients is noted in those respective dataset publications. The other portions of this framework question are not applicable. |
| Are the data suitable to answer the clinical question? | The heterogeneity and detail of the data was sufficient for our non-clinical task. |
| What computational and software resources are required for the task, and are the available resources sufficient to tackle this problem? | Hardware: eVGA nVidia Geforce 3090 (All computing primarily run through GPU)  Software: Jupyter Notebook 8.12.0, Python 3.9.16, Tensorflow 2.6.0 |
| Is the ML/AI algorithm compared to the current best technology, and against other appropriate baselines? | Yes, the performance of the machine is contrasted to similar studies performed in the discussion section of this manuscript. |
| On what basis are data accessible to other researchers? | Fully accessible. Datasets are available on Kaggle and referenced in this manuscript. |
| Are the code, software, and all other relevant parts of the prediction modelling pipeline available to others to facilitate replicability? | Fully accessible. Code has been uploaded to GitHub. Software is completely free and available for public download and use (the author utilized the software previously mentioned within an Anacondas environment). |
| Are the results generalizable to settings beyond where the system was developed? | Although no clinical generalizability is applicable, the accessibility of limited tasks to consumer grade hardware and the methods used in this study could be investigated in future work. |
| Does the model create or exacerbate inequities in healthcare by age, sex, ethnicity, or other protected characteristics? | The intent of this paper is to demonstrate consumer grade hardware need not form an absolute barrier to ML work, thus helping address inequities. However significant barriers still exist to true democratization including internet access, data science skills, challenges with patient privacy, and using ML in nuanced tasks. |
| *Other questions deemed inapplicable since the ML model will not be deployed clinically.* | |
